# Midwives’ communication with non-Swedish-speaking women giving birth: A survey from a multicultural setting in Sweden

**DOI:** 10.18332/ejm/148159

**Published:** 2022-06-16

**Authors:** Anna Akselsson, Lena Westholm, Rhonda Small, Elin Ternström

**Affiliations:** 1 Department of Health Promoting Science, Sophiahemmet University, Stockholm, Sweden; 2 Department of Women’s and Children’s Health, Karolinska Institutet, Stockholm, Sweden; 3 Judith Lumley Centre, La Trobe University, Melbourne, Australia; 4 School of Education, Health and Social Sciences, Dalarna University, Falun, Sweden

**Keywords:** birth, midwives, communication, migrant women, language barriers, inequitable care

## Abstract

**INTRODUCTION:**

The European Union faces challenges related to migration, cultural diversity and health. Immigration to Sweden has increased and a third of all women giving birth were born outside Sweden. A higher risk for negative pregnancy outcomes is seen among foreign-born women and one of the explanations given is inadequate communication. Midwives in Sweden have responsibility for normal birth. This study aimed to investigate labor ward midwives’ experiences of caring for and communicating with women who do not speak and understand the Swedish language.

**METHODS:**

A questionnaire based on the Migrant Friendly Hospital questionnaire was distributed to all 46 midwives working on the Södertälje Hospital labor and postpartum ward in 2018 and 32 completed it (70%).

**RESULTS:**

Most of the midwives thought communication and giving support to non-Swedish speaking women during birth was difficult or very difficult (n=31; 97%). The quality of the professional interpreters’ work was reported as good or very good by most of the midwives (n=31; 97%). However, the most common resource for facilitating communication during labor was an adult relative (always/often: n=25; 83%). Increased availability was the most common response for improving the interpreter service (n=22; 69%), as well as increasing the number of languages available for interpreter services (n=8; 25%).

**CONCLUSIONS:**

When women are giving birth, it is of the highest priority to improve communication between midwives and non-Swedish-speaking women. Better strategies for improving communication must be implemented in order to comply adequately with Swedish law and achieve equitable care of high quality for all, regardless of linguistic background.

## INTRODUCTION

Healthcare faces challenges related to migration, cultural diversity and health, as the European Union highlighted in the Amsterdam Declaration in 2004^1^. Healthcare systems have a responsibility to adapt, understand and meet the special needs that arise in a society with ethnic and cultural diversity, with impacts for the clinical practice of all who work in healthcare. All EU countries have ratified the international convention that healthcare is a fundamental human right for all, and this provides the foundation for the Amsterdam Declaration. In addition, challenges related to migration are also an important part of the Agenda 2030^2^, further discussed in a systematic review^3^ and recent reports by WHO^4,5^. To value diversity and acceptance of people with different backgrounds, the Migrant-friendly Hospitals (MFH) project was initiated by 12 European countries^6^. The goal was to identify the needs of migrants from different backgrounds and develop services to better meet these needs, also taking into account the disadvantages many migrants experience.

In Sweden, the Health Care law^7^ and Administrative law^8^ regulate a patient’s right to understand the information provided in healthcare. Sometimes an interpreter is required for these laws to be followed. Further, according to the Patient law^9^, information must be adapted to the recipient’s age, maturity, experience, linguistic background and other individual conditions. The person providing the information must, as far as possible, ensure that the recipient has understood the content and meaning of the information provided. The Patient law also recommends, though does not mandate, that caregivers use interpreters, but not the relatives of patients. The caregiver’s guide for Stockholm, the largest region in Sweden, emphasizes the importance of using an interpreter when necessary, to maintain patient safety^10^.

There is wide variation in levels of both education and experience among interpreters in Sweden and a clear definition of the profession is lacking. In Sweden there are 1123 interpreter authorizations (language authorizations, not individuals) and this is the only protected professional title^11,12^. Of those, 188 have special competence for interpreting in healthcare. Most of the interpreters working in healthcare are called ‘other interpreters’ and they are not obliged to have any special education or degree. The number of authorizations per year have decreased from 94 in 2018 to 27 in 2020 (due to the COVID-19 pandemic)^11,12^.

According to the World Health Organization and Swedish midwifery guidelines, a key aspect of midwives’ work during pregnancy and birth is to convey information and provide advice, support, a sense of security and continuity of care^13,14^. A midwife should have a holistic, ethical approach and care for and respect a woman’s autonomy, integrity and dignity. Further, the knowledge and experiences of women and their families should be recognized and considered in providing care.

Healthcare professionals in maternity care have reported challenges when caring for migrant women, fearing that they are not providing adequate care^15,16^. Not receiving accurate obstetric and medical histories due to women’s lack of proficiency in English is one of the challenges as is resorting to using women’s relatives or friends as interpreters^15^. Even when interpreter services are available, there can be challenges in accessing the service, often due to lack of availability at short notice outside normal working hours.

In Sweden in 2019, 30.1% of women who gave birth were born in another country and this proportion has increased gradually over several decades, from 11.1% in 1973^17^. Some groups of women who have migrated to Sweden have higher risk of negative pregnancy outcomes than those born in Sweden^18^, and problems with communication is one of the reasons^19^. Little is known about how labor ward midwives working in multicultural contexts in Sweden, manage caring for and communicating with women who do not speak and understand the Swedish language. This study aimed to explore their experiences.

## METHODS

The study was conducted in a multicultural setting, Södertälje municipality in Stockholm, Sweden, where more than half the inhabitants are of foreign background and 49.9% of women of reproductive age (15–49 years, WHO definition) were born outside Sweden^20^. A questionnaire (Supplementary file) was distributed to all 46 midwives working on the Södertälje labor and postpartum ward in March 2018. The labor and postpartum ward is on the same floor and all the midwives work in both wards. The midwives received information about the study at a staff meeting and by email. Thereafter, the study information and questionnaire were placed in all midwives’ personal mailboxes. The midwives had three weeks to submit the questionnaire and a reminder was sent by the midwife-incharge after three weeks. Completed questionnaires could be placed in a box on the ward.

The questionnaire used in this study was based on the Migrant Friendly Hospital questionnaire, a validated questionnaire often used in projects focusing on adapting healthcare to the needs of migrant populations^21^. The original questionnaire was modified for the study group and setting, after verbal agreement with the authors. The adjusted questionnaire was piloted by five midwives, a professional interpreter and a researcher not involved in the project. The final questionnaire covered respondent background information about age, work experiences, mother tongue and how often they cared for women with limited knowledge of the Swedish language. The outcome variables comprised aspects of the experience of caring for migrant women. These were: views about communicating and supporting women when giving birth; details of the most common languages encountered and the resources they used to facilitate communication; and experiences and views of the quality of interpreter services when used and suggestions for improving communication with women. The questionnaire comprised a mix of closed response and open-ended questions and the last question was an optional open-ended question to add any further comments.

All data from the questionnaires were imported to Excel by LW and AA and analyzed descriptively. We calculated respondents’ age (mean and range) and mean years in the profession. For all outcome variables, we calculated the number of responses (n) and proportion (%). The midwives answered the questionnaire anonymously and participation was voluntary. The study was approved by The Swedish Ethics Board (record number 2017/1312-31/5). There is a plan to repeat the study in the near future for comparison purposes.

## RESULTS

Of the 46 midwives who received a questionnaire, 32 responded (69.6%). The midwives’ mean age was 45 years (range: 28–65). They had worked in their profession from six months to 35 years (mean 12 years). Twenty-seven (84.4%) had Swedish as their mother tongue.

**Figure 1:**
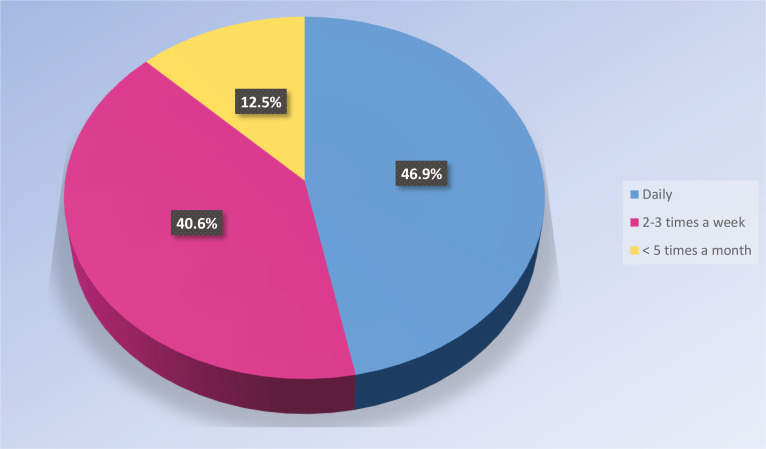
How often the participant midwives cared for women with limited knowledge of the Swedish language (N=32)

As shown in Figure 1, the midwives often cared for women with limited knowledge of the Swedish language. All the midwives (n=32; 100%) indicated that the most common language among women they cared for was Arabic. The second most common languages were Farsi (n=9; 28.1%) and Russian (n=9; 28.1%), followed by Turkish (n=7; 21.9%). Less common languages were Syrian, Polish, English, Spanish, Baltic languages, Kurdish, Tigrinja, Romanian, Bosnian Serbo-Croatian, and Urdu.

**Figure 2:**
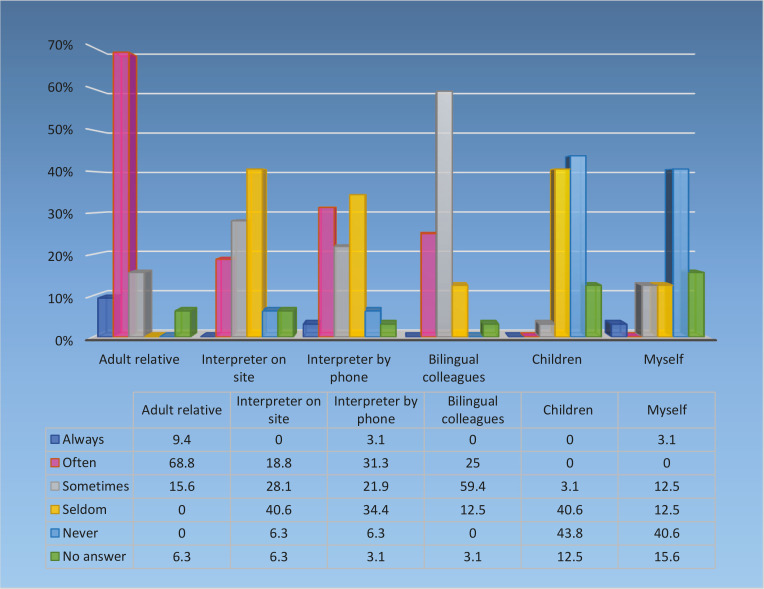
Resources midwives used to facilitate communication with non-Swedish-speaking women

Most of the responding midwives thought that communication and giving support to non-Swedish-speaking women during birth was difficult (n=22; 73.3%) or very difficult (n=7; 23.3%). One thought it was easy (3.3%) and no one thought it was very easy (missing=2). All midwives (n=32) thought a professional interpreter should facilitate communication with non-Swedish-speaking women. About half preferred an interpreter on site (n=15; 46.9%), a third by phone (n=10; 31.0%) and a fifth had no preference (n=7; 21.9%). The resources the midwives used to facilitate communication are shown in Figure 2.

Of the responding midwives (n=30; missing=2), an adult relative of the patient was the most commonly used resource for facilitating communication with non-Swedish-speaking women on the labor ward (always or often: n=25; 83.3%). Eleven midwives of 31 (missing=1) (35.5%) always or often used an interpreter by phone, while nine midwives (29.0%) mentioned in free text that they used other resources, which included community-based bilingual doulas (CBD), body language, a friend of the patient interpreting by phone, sign language or an App called ‘language in healthcare’.

In relation to the quality of the work of professional interpreters, 62.1% (n=18) judged it to be good and 34.5% (n=10) very good (missing=3). Seventeen (65.4%), reported that the interpreters were often available on time, with nine (34.6%) answering sometimes or seldom to this query (missing=6). Eighteen (66.7%) of the responding midwives (missing=5) felt certain that the interpreters conveyed medical information accurately always or often, with nine (33.3%) answering sometimes or seldom. More information about the midwives’ views on the quality of the interpreters’ work is given in Table 1.

**Table 1:** The midwives’ thoughts on the quality of the interpreters’ work

*Responses*	*You feel certain that the interpreters convey the medical information correctly (n=27) %*	*Interpreters ask you for clearance to confirm that they understood correctly (n=29) %*	*Interpreters present themselves and explain their role (n=26) %*	*Interpreters make sure you understand the message the patient tries to convey to you (n=27) %*
Never	0	0	0	0
Seldom	7.4	17.2	0	3.7
Sometimes	25.9	41.4	11.5	44.4
Often	51.9	37.9	38.5	40.7
Always	14.8	3.4	50.0	11.1

To the question ‘From your experience of using professional interpreters, in what ways can the interpreter service be improved if you feel that improvement is possible?’ where the midwife could nominate a range of alternatives, ‘Improve the interpreters' availability’ was the most common answer (n=22; 68.8%) and second was an increased number of languages available for interpreting services (n=8; 25.0%). The alternative ‘Information on how to get access to professional interpreting services’ was chosen by five midwives (15.6%) and one (3.1%) answered ‘No improvement is needed, it is good enough right now’.

Other suggestions for improvement were conveyed in free text by twelve midwives (37.5%). Most suggestions were connected to availability of interpreting services: access during weekends and nighttime, interpreters on site employed by the hospital and faster access to an interpreter when requested. Other recommendations were access to community-based bilingual doulas (CBDs) and female interpreters on the labor ward. Also, the midwives wanted more professional and accurate interpreters who do not add their own values to the interpretation. In addition, they wanted clearer guidelines from hospital management concerning the use of interpreters and education on how to communicate with an interpreter by phone.

The questionnaire concluded with an open-ended question where the midwives could convey any further thoughts about communication with non-Swedish speaking women. Nine midwives (28.1%) added information here. Comments included that it was helpful to have CBDs assisting with communication. Access to interpreters in emergency situations, a common occurrence on the labor ward, was difficult and there was often no time to find an interpreter. One midwife suggested it would be helpful with an on-call easy to access interpreter service. Another suggested that the hospital should employ an Arabic interpreter, who could be accessible at all times. As one midwife concluded: ‘It is important to create good conditions to be able to access interpreters during births while maintaining safety, integrity and respect for the mother’. If interpreters were more accessible, on site or by phone, they would be used more frequently.

## DISCUSSION

The majority of the midwives reported that communication and giving support to non-Swedish-speaking women during birth was difficult or very difficult and more access to interpreters would facilitate their work. The most common resource for communication with a non-Swedish-speaking woman during birth was an adult relative. Even when using a professional interpreter, only 67% always/often felt certain that the medical information was interpreted correctly. Areas of improvement, according to the midwives, would be increased availability of interpreters and clearer directives from hospital management that interpreters should be used during birth.

For equitable, safe and person-centered high-quality healthcare, good communication is central^22^. Patients are more satisfied with communication via a professional interpreter and mistakes associated with communication also decrease^23^. Giving birth is a significant event in a woman’s life^24^ and communication and support by the midwife are critical. Fear of childbirth is also known to be more common among foreign-born women^25^. Even though lack of a common language could not be seen as the main reason for the increased fear among foreign-born women, being understood and receiving the right information is crucial to feeling safe during pregnancy and childbirth^26^. The participating midwives in this study felt that communication and support was difficult or very difficult when assisting non-Swedish-speaking women giving birth. Using an interpreter did facilitate the communication, but the interpreter service on the labor ward was mostly impossible to schedule and therefore a reason for not using an interpreter. The midwives had a number of suggestions for improvement. Interestingly, the results showed that it was also quite common for the midwives to facilitate communication by using their own language skills or a bilingual colleague. These figures would probably have been even higher if fewer of the respondents had Swedish as their mother tongue.

It is possible that improving cultural competence could facilitate the midwives’ communication with the migrant women giving birth, as has been shown in a study in the ORAMMA project (including three European countries)^27^. The midwives’ knowledge, skills and cultural competence significantly increased after culturally sensitive maternity care training.

The findings from our study show that the availability and quality of the interpreting service needs to be improved. Most commonly, the midwives had to rely on an adult relative for help with communication which was clearly unsatisfactory and not recommended^28^. This is similar to previous findings in a study of midwives from Ireland^15^. Many disadvantages of letting adult relatives interpret have been described, such as the risk of information being filtered, the conversation being inhibited or censored, and the relative protecting the patient from unpleasant information^28^.

An on-site professional interpreter employed by the hospital was one of the midwives’ suggestions for improvement. This interpreter would preferably speak the most common language, Arabic in this setting, something also recommended in other reports and studies^28,29^. Another idea proposed by the midwives to facilitate communication during labor, was to cooperate with community-based bilingual doulas (CBDs) who accompany and support women in labor. Support in labor, both physical and emotional, from a doula has been associated with positive experiences of care and for migrant women, improved communication and information-sharing^30-32^. Interviews with women and doulas have also revealed that doulas can have a positive impact on maternal emotional wellbeing, by reducing anxiety, sadness and stress, and increasing self-esteem and self-efficacy^33^.

The Swedish National Board of Health and Welfare reported in 2016 that there is a lack of interpreters in Sweden, particularly accredited interpreters in healthcare, and the demand is continually increasing^29^. Further, assignments in healthcare may be rejected by interpreters due to lower remuneration. This can lead to serious consequences for patient safety and integrity, but also affects the efficiency and workload of the healthcare staff. These structural issues in provision of interpreter services need urgent attention, so that language barriers can be appropriately addressed, and equitable care can be given to all^34^, especially for such a significant life event as giving birth.

According to the Swedish Healthcare law^7^, the goal is good health and care on equal terms for the entire population. To be able to fulfill this goal, communication with non-Swedish-speaking women giving birth must be improved by having better access to accredited interpreters. Additionally, the Patient law^9^ stipulates a person’s right to receive information according to linguistic background. It would also likely be beneficial to have female interpreters in obstetric settings as there are such special circumstances^34^. The experiences and recommendations of health caregivers, such as the midwives in this study, should inform decision-making to improve interpreting services in healthcare settings, including review and improvements in the training of interpreters and in the training of staff to work with interpreters. More research is needed within the field of healthcare and migrant women, and the healthcare providers need to be culturally competent^3^. Further, professional interpreters should be provided at each care encounter, as outlined in a systematic review, exploring migrant women’s experiences of pregnancy, childbirth and maternity care^3^.

### Strengths and limitations

No previous Swedish study has investigated midwives’ experiences of communicating with women not fluent in the Swedish language. The study was performed in a municipality with a high prevalence of births to migrant women and therefore the participating midwives had significant experience of caring for foreign-born women. Half the inhabitants in Södertälje are of foreign background in comparison with 33.9% in the whole of Stockholm^35^. The study questionnaire had been validated and previously used in several Migrant Friendly Hospital projects^21,36^. The response rate of 70% is better than many studies of busy health professionals and the midwives who participated varied in age and number of years in the profession. All these aspects are strengths of the study.

There are some limitations, of these being a single hospital study and the findings may not be representative of the experiences of midwives in all labor wards. Yet these midwives have a great deal of experience in caring for non-Swedish-speaking women and in accessing all the available resources for communicating with them, and still they found it challenging.

Attrition bias could have affected the results as 30% chose not to complete the questionnaire and reasons for non-response were not collected. The midwives who were especially interested in the care of non-Swedish-speaking women might have been more likely to respond. However, in relation to the aim of the study, this was probably not a significant issue. Lack of time is likely to have contributed to non-response due to the high workloads in the labor ward. The availability of interpreters is more limited in rural areas of Sweden so a more representative sample of Swedish midwives would likely have increased the proportion reporting difficulties. Likewise, access to interpreters and systems for using them in areas with fewer migrant women giving birth are likely to be even more inadequate. Further research into midwives’ experiences caring for migrant women across a range of hospitals in Sweden would help to confirm the findings of the current study which suggest that significant improvements are needed.

## CONCLUSIONS

Since almost one-third of women giving birth in Sweden are born abroad, greater investment in improving communication with those not speaking Swedish is needed to fulfil expectations of equitable healthcare provision. For migrant women, giving birth may also be the first contact they have with the healthcare system and an opportunity to lay the foundations for confidence in healthcare in their new country. For this to occur successfully, and to reduce known adverse pregnancy outcomes among migrant women, good communication is vital. Midwives are key providers of maternity care and must be supported with the necessary resources to provide excellent and equitable care for all women and their families, including accessible and high-quality interpreting services.

## Data Availability

The data supporting this research are available from the authors on reasonable request.
